# Safety of Occlutech Septal Occluder ACCELL Flex II for Transcatheter Closure of Secundum Atrial Septal Defects in Children: A Long-Term Follow-Up

**DOI:** 10.1155/2022/8886813

**Published:** 2022-01-04

**Authors:** Amal M. El-Sisi, Sonia A. El-Saiedi, Rasha Ammar, Asmaa Abdelhameed, Ziyad M. Hijazi, Mohammed M. Soliman

**Affiliations:** ^1^Cairo University-Pediatric Department-Cardiology Division, Cairo, Egypt; ^2^Ministry of Health Hospitals, Cairo, Egypt; ^3^Sidra Heart Center, Sidra Medicine& Weill Cornell Medical College, Doha, Qatar

## Abstract

**Objectives:**

To assess the long-term safety and efficacy of the Occlutech® ACCELL® Flex II device used for atrial septal defect (ASD) closure. This device differs from the regular device by having two very thin patches that are made of polyethylene terephthalate (PET). These patches enhance faster sealing of the defect.

**Background:**

Transcatheter closure has become the method of choice to manage most patients with secundum ASDs. There are different types of devices. The regular Occlutech device used to close an ASD is called the Occlutech Figulla Flex II. A newer modification of this device (Occlutech® ACCELL® Flex II) has been designed to eliminate/reduce thrombus formation and to enhance faster sealing.

**Methods:**

Thirty patients were followed up after occlusion of secundum ASD using the Occlutech® ACCELL® Flex II Device. The follow-up period ranged from 5.2–5.5 years with median of 5.3 years. Detailed history and full clinical examination, twelve-lead electrocardiogram (ECG), plain chest radiograph, and full 2D transthoracic echocardiography (TTE) were performed at discharge, at one month, six months, and yearly thereafter.

**Results:**

The mean age of the study group at the last follow-up was 10.4 ± 4.6 years, with 63.3% (nineteen patients) females. There were no residual shunts or complications encountered immediately after the procedure and at the latest follow-up.

**Conclusion:**

This study confirmed the transcatheter closure (TCC) of secundum ASDs using the Occlutech® ACCELL® Flex II device to be safe and effective with no complications detected in children and adolescents.

## 1. Introduction

Atrial septal defects (ASDs) are common and account for about 6–10% of all congenital heart lesions [1].

Due to its efficacy and reliability, transcatheter device closure of a secundum ASDs had emerged to be the principal choice for management of secundum ASDs. It results in less complications and reduced hospitalization, as compared to open surgical ASD closure [2–4].

The inherent advantages of transcatheter closure (TCC) of ASDs include the absence of a thoracotomy incision, no intensive care unit admission, less psychological impact, shorter hospital stay, and less need for blood transfusion. The absence of a myocardial scar may decrease the incidence of incisional dysrhythmias [1].

The ideal device should be repositionable, retrievable prior to release, and should facilitate complete closure with minimal risk of embolization. Additionally, it should facilitate rapid endothelialization and be biocompatible and suitable for treating a wide range of defect characteristics. The standard Occlutech Figulla® Flex II device (FSO; Occlutech GmbH, Jena, Germany) is a nitinol double-disk occluder that possesses these characteristics and is used frequently for closure of secundum ASDs [5–9].

The device consists of two discs with a larger intermediate waist. Inside each of the discs, there is a Polyethylene (PET) patch, to support the immediate closure. This helps stop the blood going through the meshwork of the device. Occlutech unique patented braiding technology allows the device to be manufactured without a left-sided hub. The absence of a left disc hub minimizes the risk of thrombus formation, and it confers softness to the left disc; thus, we believe it minimizes the incidence of device erosion. The device is pushed through the delivery catheter across the defect, then one disc is deployed in the left atrium and the waist is deployed to “stent,” the defect itself, and finally, the right atrial disc is deployed in the right atrium. The procedure is best performed under echocardiographic guidance (transesophageal or transthoracic or intracardiac echocardiographic) [10].

Thrombus formation on atrial septal occluders is rare, but it is a potential serious complication that could lead to an embolic event. A newer modification of this device (Occlutech® ACCELL® Flex II) has been designed to eliminate/reduce this potential complication. The new device has modification to the standard Occlutech Figulla device which includes adding two very thin patches to the regular device made of polyethelene terephtalate (PET) to enhance faster sealing of the defect [11].

## 2. Patients and Methods

This is a descriptive retrospective study that included all patients who had secundum ASD closure by using the Occlutech® ACCELL® Flex II device (Occlutech International AB Rönnowsgatan 8 SE- 252 25 Helsingborg, Sweden). The study was a single-arm, open-label, nonrandomized pilot clinical trial performed at Cairo University Children Hospital. The Occlutech® ACCELL® Flex II device risk analysis (per ISO 14971) of the medical device(s) had been conducted and can be referenced to the Clinical Evaluation report, doc id: D09C04A01 [11]. All potential risks have been minimized or eliminated through appropriate design control and confirmed by preclinical bench, laboratory, and animal testing.

The procedures that were conducted as part of this clinical trial were standard procedures, and all investigators were familiar with the use of the device. There was no added risk on the patients participating in the trial. All patients were assessed during their regular follow-up visits in the outpatient cardiology clinic. The procedures were performed from September 2014 to January 2015.

Inclusion criteria: all patients who underwent ASD closure by using the Occlutech® ACCELL® Flex II device met all inclusion criteria similar to the standard device. Exclusion criteria: again, any patient that did not meet the inclusion criteria for the standard device was excluded from the study.

**Figure 1:**
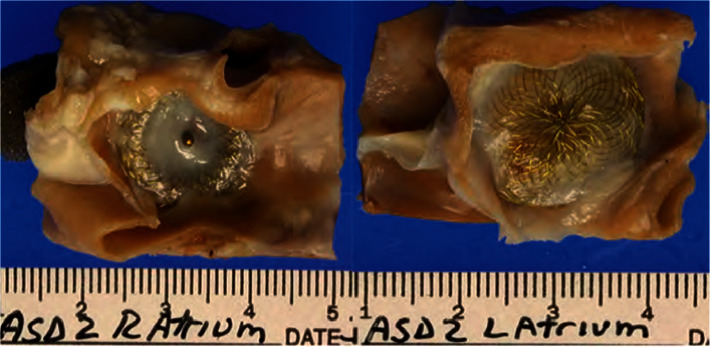
ASD device uncoated.

**Figure 2:**
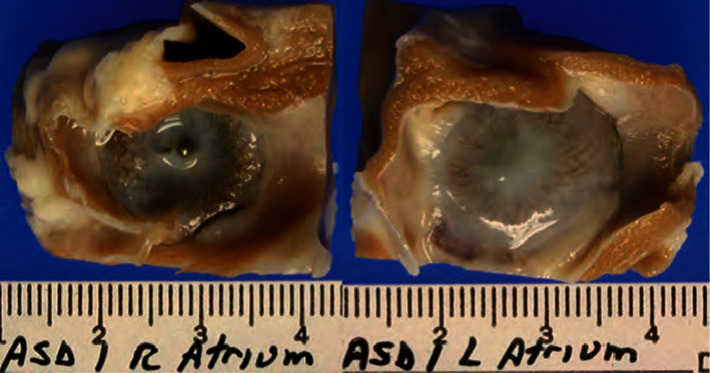
ASD device coated.

The Occlutech® ACCELL® Flex II ASD device consists of a nitinol wire mesh with shape-memory properties. A flexible waist connects the two small retention discs, which fully adjust to the atrial septum after leaving the delivery sheath. Two very thin patches made of PET ensure faster sealing of the defect in the atrial septum while optimizing ingrowth of tissue. The device is coated with a constant polymer. The polymer for coating of the device is effective in enhancing the adhesion of endothelial cells onto surfaces which provide rapid endothelialization on the device surface. The faster endothelialization of the device helps anchoring the device to the atrial septum and sealing of the residual shunt through the device which provides superior occlusion of the defect and minimizes the risk for left-to-right shunt after device implantation. The sponsor performed a number of in vitro and in vivo preclinical tests to establish the safety of coated Flex II ASD occluders (ACCELL® Flex II ASD occluders). The results demonstrated that ACCELL® coated occluders and/or materials are biocompatible, tolerable, and safe. In addition, these tests demonstrated that ACCELL® can enhance endothelialization of implanted devices. ACCELL® is a proprietary biocompatible polymer composition developed by Hydromer, Inc. (Branchburg, NJ) to provide a nondrug mechanism for enhancing cellular attachment. ACCELL® Flex II occluders are coated with ACCELL®. In summary, the results from this study demonstrated that ACCELL® Flex II occluders were safe and showed similar performance as uncoated occluders when implanted in pigs' hearts. Figures 1 and 2 show the difference in animal studies between coated and noncoated devices after ninety days.

TCC of secundum ASDs was performed under general anaesthesia with continuous transesophageal echocardiograghic (TEE) guidance in all patients [3]. Device size was chosen to be 2–4 mm larger than the largest ASD diameter by color Doppler echocardiography. Antiaggregation therapy with aspirin, 5 mg/kg/day orally, was prescribed for six months after the procedure [3].

All patients had the following: detailed history, full clinical examination, twelve-lead surface electrocardiogram (ECG), plain chest radiograph, and complete transthoracic echocardiography (TTE) using (GE Medical Systems, Norway) Vivid 5 and 7 ultrasound machines with 3 and 5 MHz probes. Echocardiographic examinations consisted of (a) conventional echo-Doppler, M-mode measurements, 2D, pulse wave (PW), continuous wave (CW), and color Doppler blood flow velocity measurements of the heart valves; (b) tissue Doppler: pulsed wave tissue Doppler imaging (PW-TDI) measures included systolic (S′) and diastolic (E′, A′, E′/A′ ratio); calculation of the global myocardial performance index (MPI) (Tei index) of both the right ventricle (RV) and left ventricle (LV) according to the guidelines of the American Society of Echocardiography [12]. Residual shunt by color Doppler was graded as trivial shunt: jet width <1 mm; small shunt width 1-2 mm; moderate shunt width 2–4 mm; and large shunt width >4 mm) [3].

### 2.1. Statistical Analysis

Data were entered and statistically analyzed on the Statistical Package of Social Science Software program, version 23 (IBM SPSS Statistics for Windows, Version 23.0. Armonk, NY: IBM Corp.). Data are presented as mean and standard deviation for quantitative variables if the data are normally distributed or as the median and interquartile range if the data are not normally distributed. For the qualitative variables, data are presented as frequency and percentage. Test for data normality was conducted using inspection of the histogram.

### 2.2. Ethical Statement

All investigations involving human participants were performed in accordance with the ethical standards of the institutional and national research ethics committees and with the 1964 Helsinki Declaration and its later amendments. The study was approved by the ethical committee at Cairo University Children Hospital.

## 3. Results

The study included long-term follow-up (FU) of 30 patients who underwent transcatheter occlusion of secundum ASDs using the Occlutech® ACCELL® Flex II Device. The age, height, weight, and body surface area were at the procedure and last FU as follows, respectively: regarding age, range was 2–14 years and 5–18 years and median age was 3.3 and 7 years; the range of weight was 8–65 and (15.5–75 and median was 14.5 and 17.5 kilograms (Kg); the range of height was 65–170 and 113–175 and median 90 and 125 cm; and the range of body surface area was 0.45–1.3 and 0.76–2 and median 0.6 and 1.2 m^2^.

ASD size range is 12–30 mm with a median of 14 mm, ASD device size range was 10–33 mm with a median of 15 mm, and the size of the delivery sheath ranges from 8–14 Fr with a median of 10 Fr. The range of follow-up was 5.2–5.5 years with a median of 5.3 years (Table 1).

Nineteen patients (63.3%) were females, and Qp/Qs was more than 1.5 in all cases. There were no deaths, no endocarditis, and no rhythm disturbances or other complications during the whole follow-up period. Subjects with frequent respiratory infections had no significant recurrences. One patient had a complaint of chest pain (3.3%) which was musculoskeletal relieved with analgesics, and two had breathlessness on exercise (6.7%) which was related to concurrent anemia (Table 2).

All cases showed normal chest radiograph for device position and no other abnormalities. Two patients had sinus tachycardia, and the other patients showed normal ECG with no heart block or any arrhythmias or right ventricle enlargement. Two patients had mild mitral regurgitation, and one patient had mild aortic regurgitation that persisted till the last FU. Unfortunately, the echocardiograms before the procedure did not mention the presence of mitral or aortic regurgitation in these patients. Eleven patients had mild tricuspid regurgitation from which pulmonary pressure was calculated, and five had mild pulmonary regurgitation at the last FU. There was no evidence of pulmonary hypertension in any of the cases at the last FU. Echocardiographic measurements and Z-scores of the left ventricle (LV), right ventricle (RV), and PW of the mitral and tricuspid valves are within the normal range at the last FU (Table 3).

Table 4 showed the tissue Doppler imaging data of our patients at the last follow-up, but unfortunately, the data before the procedure and immediately after it were not available.

All cases had no residual shunt through the device, and they all showed good alignment of device position with the interatrial septum (IAS); all patients had no evidence of thrombosis and compression on the superior vena cava (SVC), inferior vena cava (IVC), pulmonary veins, or any adjacent structure.

## 4. Discussion

Many devices are available for ASD occlusion, combining different properties, such as the ability to recapture and redeploy the device within the delivery sheath, the self-centering mechanism to simplify and achieve good positioning, leading to a high occlusion rate, and finally, a wide availability of sizes to close small to large defects. The Amplatzer® Septal Occluder (ASO; Abbott) has been used most widely for over 20 years, with favorable follow-up results. More recently, the Figulla® ASD Occluder (FSO; Occlutech GmbH, Jena, Germany) has been developed, with structural innovations [5].

In comparison with the Amplatzer Septal Occluder (ASO), the Figulla Occlutech device (FSO) has several advantages. It has no left atrial hub minimizing the risk of clot formation and providing softness to the left atrial disc; thus, we believe it minimizes the risk of erosion. It has a unique delivery system that provides unique tilting abilities between the delivery cable and right atrial disc, thus allowing good alignment of the discs across the defect and minimizing left atrial disc prolapse into the right atrium [5].

In our study, we followed up 30 patients for five years after occlusion of their ASDs using the new Occlutech® ACCELL® Flex II Device in an effort to assess the safety and efficacy of this device.

Despite the use of relatively large sheath sizes, there were no reported vascular complications immediately after catheterization or later on in follow-up.

The delivery sheath profile of Occlutech devices is relatively larger than that of others because of the bioptome-like delivery cable and the pivoting ball and socket design of the right atrial disc, but this was not associated also with greater vascular complications in Kenny et al.'s study [13].

Two patients (6.6%) had dyspnea on exertion, and one patient (3.3%) had palpitation All these symptoms resolved after the first 6 months.. The remainders were asymptomatic.

The reported complications of TCC of secundum ASDs are rare and mostly early in the vast majority of patients. In Fischer et al.'s study, there were no immediate complications. They only reported arrhythmia early after Amplatzer Septal Occluder (ASO) implantation was an increase in supraventricular and ventricular ectopy immediately and 24 h after the procedure [14].

In Nurun's study, embolization of the device to the right ventricle was experienced in 1% of cases and transient arrhythmia was observed in 0.39% of cases. ECG finding of ST changes due to air embolism was experienced in 1.2% of cases; only one patient had cardiac perforations, no one had major bleeding, and no one had sudden death [15].

In Pedra et al.'s study, there were 2 instances of device embolization to the aorta with retrieval and new implantation and no other complications [16].

In the study of Bahajati et al., there was a minor complication including transient sinus tachycardia in 10 patients and paroxysmal supraventricular tachycardia in two patients, which successfully terminated with adenosine and verapamil [1].

In Kenny et al.'s study, there was an overall rate of complications of 5.6% including residual shunts in 4 patients, significant groin hematomas in 2 patients, and only one patient with temporary atrial arrhythmia. This supports that Occlutech Figulla flex II devices are safe with low incidence of complications [13].

All our cases had no arrhythmias, no residual shunt, and good alignment of device position with the interatrial septum (IAS), no evidence of thrombosis, and no compression on the superior vena cava (SVC), inferior vena cava (IVC), pulmonary veins, or any adjacent structure. There was no erosion in any of the cases.

## 5. Conclusions

Our study shows that transcatheter closure of secundum ASDs using the Occlutech® ACCELL® Flex II Device was safe and effective in children and adolescents. Our patients had excellent long-term outcomes, and no long-term complications could be detected. The ACCELL coating on the device may enhance endothelialization of the occluder and, therefore, may minimize the risk of thrombus formation on the occluder.

### 5.1. Limitations

The limitation include the following: small number of patients, the Accell device did not proceed to CE marking, and there were no adult patients in this clinical trial to test the device in a better way.

## Figures and Tables

**Table 1:** Demographic characteristics of the study group at the procedure and last FU.

	Range	Median
Age at the procedure (years)	2–14.0	3.3
Age at the FU (years)	5–18	7
Weight at the procedure (Kg)	8–65	14.5
Weight at the FU	15.5–75	17.5
Height at the procedure(cm)	65–170	90.0
Height at the FU	113–175	125
BSA at the procedure (m2)	0.45–1.3	0.6
BSA at the FU (m2)	0.76–2	1.2
ASD size (mm)	12.0–30.0	14.0
ASD device size (mm)	10.0–33.0	15
Sheath size (Fr)	8–14	10
FU (years)	5.2–5.5	5.3
HR (beats/min)	60–100	89
Systolic BP (mmHG)	100–120	105
Diastolic BP (mmHg)	60–85	71

FU: follow-up, Kg: kilogram, CM: centimeters, ASD: atrial septal defect, BSA: body surface area, HR: heart rate; BP: blood pressure, Fr: French.

**Table 2:** Frequency of different symptoms and vital signs among the study group.

Variables	Number (*n* = 30)	%
Female	19	63.3
Qp/Qs >1.5 at the catheter	30	100
Residual shunt immediately after the procedure	0	0
Residual shunt at the last FU	0	0
Palpitation	0	0
Chest pain	1	3.3
Breathlessness on exercise	2	6.7
Signs of heart failure	0	0

Qp; pulmonary flow, Qs: systemic flow, FU: follow-up.

**Table 3:** Description of 2D echo, M-mode, PW, and CW measurements and Z-score among the study group.

Variables	Range	Median
LVEDD (cm)	3.2–5.3	3.8
LVEDD Z-score	−2–1.33	−0.06
LVESD (cm)	2–3	2.3
LVESD Z-score	−2.7–1.2	−0.065
IVSD (cm)	0.40–1.2	0.6
PWD (cm)	0.4–1.2	0.6
FS %	0.35–0.51	0.4 (40%)
EF %	0.61–0.83	0.73 (73%)
RVEDD (cm)	1.4–3.3	2.1
RVEDD Z-score	−1.3–2.4	0.07
MAPSE (cm)	1.3–2.6	1.45
TAPSE (cm)	1.8–2.7	2
MV-E	0.53–1.16	0.86
MV-E Z-score	−2.9–1.04	−1.4
MV-A	0.21–2.63	0.4
MV-A Z-score	−2–2.9	−0.14
MV E/A ratio	1.43–2.63	1.97
MV-E/A Z-score	−1.7–0.9	−0.59
TV-E	0.37–0.77	0.53
TV-E Z-score	−0.91–2.5	0.78
TV-A	0.26–0.5	0.34
TV-A Z-score	−2.4–1.7	−1.67
TV E/A ratio	1.15–1.95	1.56
TV E/A Z-score	−1.5–1.1	0.2
PAP (mmHG) in 11 cases	16–33	27

LVED/SD: left ventricle end diastolic/systolic diameter, IVSD: interventricular septum diameter, PWD: post wall diameter, FS: fraction shortening, EF: ejection fraction, RV: right ventricle, MAPSE/TAPSE: mitral/tricuspid annular plane systolic excursion, MV: mitral valve, TV: tricuspid valve, PAP: pulmonary artery pressure.

**Table 4:** TDI data of RV and LV at the last FU.

TDI	Right ventricle mean ± SD	Left ventricle mean ± SD
E` (cm/s)	14.4 ± 2.8	13.88 ± 2.2
A` (cm/s)	9.4 ± 3.9	7.05 ± 1.8
S` (cm/s)	11.9 ± 2.58	9.2 ± 2.8
E/E` ratio	5.52 ± 2	5.97 ± 2.8
MPI	0.46 ± 12	0.47 ± 14

TDI: tissue Doppler imaging; MPI: myocardial performance index.

## Data Availability

This is a descriptive retrospective study that included all patients who had secundum ASD closure by using the Occlutech® ACCELL® Flex II device (Occlutech International AB Rönnowsgatan 8 SE- 252 25 Helsingborg, Sweden). The study was a single-arm, open-label, nonrandomized pilot clinical trial performed at Cairo University Children Hospital. The Occlutech®ACCELL® Flex II device risk analysis (per ISO 14971) of the medical device(s) had been conducted and can be referenced to the Clinical Evaluation report, doc id: D09C04A01 [11]. All potential risks have been minimized or eliminated through appropriate design control and confirmed by preclinical bench, laboratory, and animal testing.

## Abbreviations

ASD: Atrial septal defect
ASO: Amplatzer septal occluder
BSA: Body surface area
ECG: Electrocardiogram
FU: Follow-up
Kg: Kilogram
IAS: Interatrial septum
IVC: Inferior vena cava
MPI: Myocardial performance index
LV: Left ventricle
RV: Right ventricle
SVC: Superior vena cava
TCC: Transcatheter closure
TEE: Transesophageal echocardiography
TDI: Tissue doppler imaging
TTE: Transthoracic echocardiography
PET: Polyethelene terephtalate.
